# Effect of Fermented Milk Supplemented with Nisin or Plantaricin Q7 on Inflammatory Factors and Gut Microbiota in Mice

**DOI:** 10.3390/nu16050680

**Published:** 2024-02-28

**Authors:** Yisuo Liu, Yushan Bu, Jiayuan Cao, Yinxue Liu, Tai Zhang, Linlin Hao, Huaxi Yi

**Affiliations:** 1 State Key Laboratory of Marine Food Processing & Safety Control, College of Food Science and Engineering, Ocean University of China, Qingdao 266000, China; liuyisuo@stu.ouc.edu.cn (Y.L.); buyushan@stu.ouc.edu.cn (Y.B.); 11230711035@stu.ouc.edu.cn (J.C.); liuyinxue@stu.ouc.edu.cn (Y.L.); tyzhang@stu.ouc.edu.cn (T.Z.); hll6446@stu.ouc.edu.cn (L.H.); 2 Food Laboratory of Zhongyuan, Luohe 462300, China

**Keywords:** bacteriocin, nisin, plantaricin Q7, fermented milk, gut microbiota, inflammatory cytokines

## Abstract

Lactic-acid-bacteria-derived bacteriocins are used as food biological preservatives widely. Little information is available on the impact of bacteriocin intake with food on gut microbiota in vivo. In this study, the effects of fermented milk supplemented with nisin (FM-nisin) or plantaricin Q7 (FM-Q7) from *Lactiplantibacillus plantarum* Q7 on inflammatory factors and the gut microbiota of mice were investigated. The results showed that FM-nisin or FM-Q7 up-regulated IFN-γ and down-regulated IL-17 and IL-12 in serum significantly. FM-nisin down-regulated TNF-α and IL-10 while FM-Q7 up-regulated them. The results of 16S rRNA gene sequence analysis suggested that the gut microbiome in mice was changed by FM-nisin or FM-Q7. The *Firmicutes/Bacteroides* ratio was reduced significantly in both groups. It was observed that the volume of *Akkermansia_Muciniphila* was significantly reduced whereas those of *Lachnospiraceae* and *Ruminococcaceae* were increased. The total number of short-chain fatty acids (SCFAs) in the mouse feces of the FM-nisin group and FM-Q7 group was increased. The content of acetic acid was increased while the butyric acid content was decreased significantly. These findings indicated that FM-nisin or FM-Q7 could stimulate the inflammation response and alter gut microbiota and metabolic components in mice. Further in-depth study is needed to determine the impact of FM-nisin or FM-Q7 on the host’s health.

## 1. Introduction

Food preservatives have become crucial additives in the food industry. Current research on food preservatives focuses on analyzing their safety and efficacy and developing novel additives [1]. Food preservatives are used to extend the storage period of food by inhibiting food spoilage organisms. Food preservatives can be classified into synthetic and naturally derived depending on the source [2]. Due to their natural and non-toxic properties, biological preservatives will be the preferred alternative to synthetic chemical preservatives in the future. Bacteriocins produced by lactic acid bacteria are typical biological preservatives that are small peptides with bacteriostatic activity [3]. Bacteriocins are currently widely used as food biological preservatives and antibiotic substitutes in food storage and animal husbandry, among which nisin is the most representative and the effect of which is notable in the processing of dairy products [4,5].

It was reported, in some studies, that bacteriocins could inhibit spoilage and pathogenic bacteria in foods and hosts [3,6]. PediocinPA-1 and ABP-118 produced by *Pediococcus acidilactici* effectively inhibited *Listeria monocytogenes* [7]. This showed that the bacteriocins Av-CD291.1 and Av-CD291.2 from *Pseudomonas aeruginos* could inhibit the colonization of *Clostridium difficile* in the gut of a host [8]. The addition of enterocin A and enterocin B produced by *Enterococcus faecium* to meat products effectively restrained the infection of *Listeria monocytogenes* [9]. It was demonstrated that enterocin AS-48 from *Enterococcus faecium* had a synergistic effect with lysozymes in controlling *Bacillus cereus* and *Staphylococcus aureus* [10]. Umu et al. found that five *Lactobacillus* species producing type-II bacteriocin altered the gut microbiome structure at the genus level [11]. Guinane et al. pointed out that the effect of bacteriocins on the structure of the host microbiota varied depending on their species and inhibitory spectrum [3].

Currently, nisin and pediocin have been used as food preservatives successfully, and many new bacteriocins have been reported [12]. A lot of research has focused on the toxic effects of bacteriocins on humans and animals to determine their safety or assess them qualitatively and quantitatively in terms of content and structure [13]. The gut microbiome is significant to the host’s immune system and health. The structure and the composition of the gut microbiome are closely related to the host’s health status. In fact, bacteriocins with excellent antimicrobial activity have been added in food processing as food additives and ingested with food in the host gut. Presently, most studies focus on the effect of the pure bacteriocins on gut microbiota while there is little research on the impact of food supplemented with bacteriocins on gut microbiota. Therefore, it is necessary to determine whether bacteriocin intake with food can specifically regulate gut microbiota and affect host health [14,15]. In this study, the effects of fermented milk supplemented with nisin or plantaricin Q7 from *Lactiplantibacillus plantarum* Q7 on the inflammatory and gut microbiota of mice were investigated, which provided a theoretical basis to incorporate gut microbiota as part of an evaluation index for food preservatives.

## 2. Materials and Methods

### 2.1. Preparation of Bacteriocin-Added Fermented Milk

Plantaricin Q7 was extracted from the liquid culture medium of *Lactiplantibacillus plantarum* Q7 (GenBank: CP019712-16) [16]. Skimmed milk powder was used as raw material for the preparation of fermented milk. Fermented milk samples were prepared with some modifications by referring to the method of Wang et al. [17]. Skimmed milk was heated to 60 °C with magnetic stirring, and sucrose (60 mg mL^−1^, *w*/*v*) was slowly added, then held at 95 °C for 15 min and cooled down with ice water. When the temperature was lowered to 43 °C, the yogurt starter (Beijing Doit Biotechnology Co., Ltd., Beijing, China) was inoculated (0.1 mg mL^−1^, *w*/*v*) in an aseptic environment and fermented for 6–8 h at 43°C. Finally, nisin and plantaricin Q7 (0.05 g/kg) were added.

### 2.2. Construction of Animal Models

Six-week-old male C57BL/6J mice (Beijing Viton Lihua Laboratory Animal Technology Co., Ltd., Beijing, China) [Animal Qualification Certificate No. SCXK (Beijing) 2014-0001] were acclimatized and fed for 1 week in an animal house with safety standards and given adequate amounts of water and food. Ethics approval for all experiments was obtained from the Animal Ethics Committee of Ocean University of China (Grant No: SPXY2022030803). According to the initial body weight, the mice were randomly divided into three groups, namely fermented milk group (FM), fermented milk + nisin group (FM-Nisin), and fermented milk + plantaricin Q7 group (FM-Q7). Each group had 12 mice. All mice were given 200 μL of fermented milk samples per day for 4 weeks, and body weight, water intake, and food intake were recorded weekly for each mouse.

### 2.3. Determination of Organ Coefficients in Mice

During the four-week feeding period, the weight of mice was recorded every 7 days and the food and water intake were recorded every 3 days. The liver, kidney, spleen, and ileocecal valve with intact shapes in the body were removed and weighed. The mass-to-body ratio of each organ of the mice was calculated based on the mass of the viscera and the corresponding body weight of the mice. The corresponding calculation formula was Organ index (%) = M_Organ_/M_Body_ × 100% [18].

### 2.4. Enzyme-Linked Immunosorbent Assay for Cytokines

Plasma was obtained from the mice’s eyes and placed for 2 h. The serum was obtained through centrifugation at 4000 rpm under 4 °C for 40 min. The absorbance values of TGF-β1, IFN-γ, TNF-α, IL-10, IL-12, and IL-17 in serum were detected by using ELISA kits (Suzhou Calvin Biotechnology Co., Ltd., Suzhou, China).

### 2.5. 16S rRNA Gene Sequencing 

This was followed by the previous report with minor modifications [19]. Fecal DNA extraction and high-throughput sequencing were performed by Personal Biotechnology Co., Ltd. (Shanghai, China). Genomic DNA was extracted from mouse feces using the Fast DNA SPIN extraction kit (MP Biomedicals, Santa Ana, CA, USA). The V3-V4 region of the bacterial 16S rDNA gene was selected for amplification. The library was characterized based on the TruSeq Nano DNA Library Preparation Kit from Illumina and sequenced using Illumina’s NovaSeq platform. Species taxonomic and classification annotations were obtained through comparison of Usearch with the Silva database (SU132). Based on these results, alpha diversity, beta diversity, species composition, species comparisons, and species differences were analyzed. Association analysis was conducted between microbial species and SCFAs to preliminarily explain the interaction between gut microbiota, nisin, and plantaricin Q7.

### 2.6. Detection of SCFAs by GC-MS

A 30 mg sample of feces was weighed at 1.5 mL in sterile, enzymatic centrifuge tubes, and 20 μL of 2-ethylbutyric acid was added to the tubes for inner control. The samples were ground in a grinder for 2 min. Oxidation was performed in a shaky incubator at 37 °C for 90 min. Quantities of 80 μL of BSTFA derivatization reagent and 20 μL of n-hexane were added to the reaction solution and underwent swirling and jittering for 2 min, followed by another 60 min at 37 °C. A quantity of 1 μL of the derivatized extract was injected into a GC-MS system (7000E Triple Quadrupole GS/MS EI/CI Bundle, Agilent Technologies, Palo Alto, CA, USA) in splitless mode for analysis. HP-FFAP column (30 m × 250 pm × 0.25 m; Agilent Technologies, Palo Alto, CA, USA) was used for chromatographic analysis. The program was as follows: hold for 2 min at 90 °C, increase to 150 °C at 12 °C/min, increase to 220 °C at 20 °C/min, and hold for 4.5 min. The run time was 15 min. Helium was the carrier gas, with a flow rate of 1 mL/min, and splitless mode was used for sample injection.

### 2.7. Statistical Analysis of Data

Data obtained from the experiment were processed using IBM SPSS Statistics 22.0 software. All values are expressed as means ± standard deviations (SDs). Comparisons of various anatomical measurements were made through two-way analysis of variance (ANOVA). The LSD method was selected if the variance was consistent, and Dunnett’s T3 procedure was used if the variance was non-uniform. *p* < 0.05 was considered statistically significant difference in treatment results. GraphPad Prism 9.5.1 (GraphPad Software, La Jolla, CA, USA) software was imported to plot the analyzed results.

## 3. Results

### 3.1. Effects of FM-Nisin and FM-Q7 on Mouse Physiological Index and Organ Coefficients

**Figure 1:**
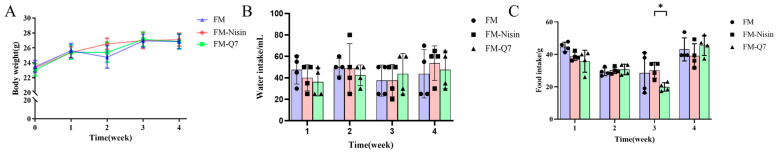
Effects of FM-Nisin and FM-Q7 on mouse physiological index. (**A**) Body weight; (**B**) water intake; (**C**) food intake (* *p* < 0.05).

The effects of fermented milk supplemented with nisin or plantaricin Q7 on body weight, food, and water intake were investigated. The results showed that the mice’s body weights were increased in the FM group, FM-Nisin group, and FM-Q7 group, and there was no significant difference among the three groups (Figure 1A). It was observed that adding nisin or plantaricin Q7 to the fermented milk did not negatively affect the food and water intake of mice compared to the FM group (Figure 1B,C). Compared with the organ coefficient of the FM group, there was no significant difference in the FM-Q7 group mice while there was a change in the FM-Nisin group (Table 1). The liver and spleen coefficients of the FM-Nisin group were higher than those of the FM group.

### 3.2. Effects of FM-Nisin and FM-Q7 on the Expression of Inflammatory Factors

**Figure 2:**
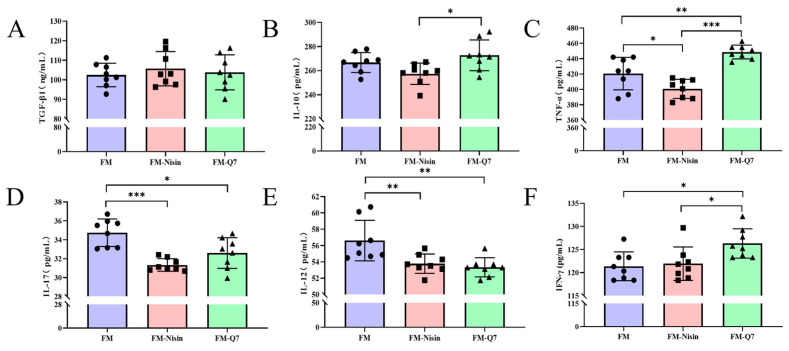
Effects of FM-Nisin and FM-Q7 on the expression of inflammatory factors. (**A**) TGF-β1; (**B**) IL-10; (**C**) TNF-α; (**D**) IL-17; (**E**) IL-12; (**F**) IFN-γ. Data are presented as means ± SDs (n = 8). * *p* < 0.05, ** *p* < 0.01 and *** *p* < 0.001.

The mouse serum was analyzed using the ELISA kit to detect the effects of FM-Nisin and FM-Q7 on the expression of the inflammatory factors TGF-β1, IFN-γ, IL-17, IL-12, TNF-α, and IL-10. It was found that there was no significant change in TGF-β1 among the three groups (Figure 2A) while the expression level of IL-10 in FM-Q7 was significantly higher than that in the FM-Nisin group (Figure 2B). In comparison to the FM group, the ingestion of FM-Q7 significantly up-regulated IFN-γ and TNF-α (Figure 2B,C) and down-regulated IL-17 and IL-12 significantly (Figure 2D,E). Mice in FM-Nisin group showed a significant down-regulation of TNF-α, IL-17, and IL-12 in serum (Figure 2C–E). Overall, the oral administration of FM-Nisin and FM-Q7 resulted in the simultaneous adjustment of the expression levels of pro-inflammatory and anti-inflammatory factors.

### 3.3. Effects of FM-Nisin and FM-Q7 on Gut Microbiota

**Figure 3:**
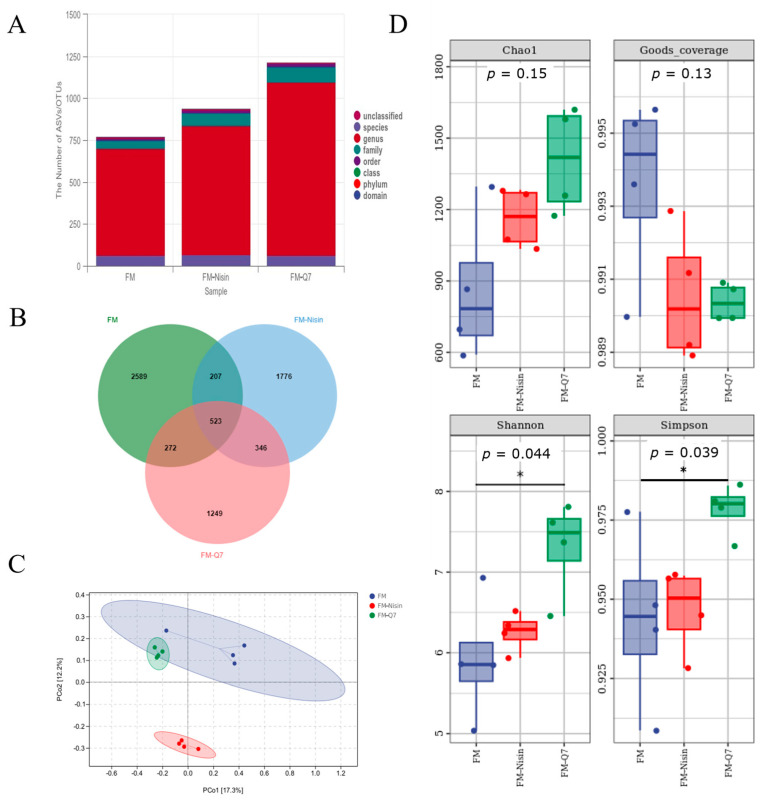
Effects of FM-Nisin and FM-Q7 on gut microbiota composition in mice. (**A**) Species classification annotations. (**B**) Venn diagrams of ASVs/OTUs. (**C**) β-diversity assessed via PCoA. (**D**) α-diversity calculated through QIIME2. * *p* < 0.05.

Alpha diversity and beta diversity were used to represent species richness between and within groups, respectively. Figure 3 illustrates the effects of FM-Nisin and FM-Q7 on gut microbiota. As depicted in Figure 3D, the Simpson index and Shannon index of plantaricin Q7 group showed substantial rises when exposed to FM-Nisin and FM-Q7 (*p* < 0.05). FM-Q7 had a more significant influence on the number and variety of host gut microorganisms. This result aligned with the rise in the OTU counts (Figure 3A,B). In terms of beta diversity (Figure 3C), the FM-Nisin group showed significant positional differences compared to the FM-Q7 group and FM group. Overall, the intake of FM-Nisin and FM-Q7 could promote the variety and number of host gut microbiota.

**Figure 4:**
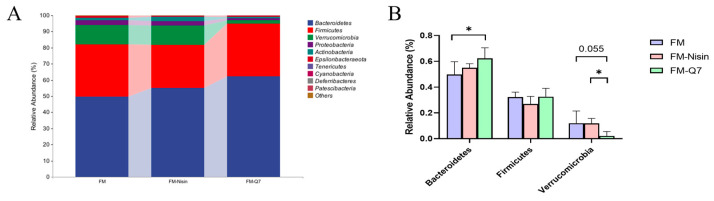
The relative abundance of different bacteria at the phylum level. (**A**) Microbial community bar plot by phylum. (**B**) The proportions of *Bacteroidetes*, *Firmicutes*, and *Verrucomicrobia*. * *p* < 0.05.

The colonic contents of C57BL/6J mice were gathered and examined to understand the microbial species composition. At the phylum level, the microbial communities of all groups predominantly comprised *Bacteroidetes*, *Firmicutes*, *Verrucomicrobia*, *Proteobacteria*, *Actinobacteria*, *Epsilonbacteraeota*, *Deferribacteres*, *Cyanobacteria*, and *Tenericutes* (Figure 4A). Obvious alterations in *Bacteroidetes*, *Firmicutes*, and *Verrucomicrobia* were observed (Figure 4B). After the intake of FM-Q7, there was a notable rise in the occurrence of *Bacteroidetes* species and SCFAs-producing bacteria while a substantial decline of *Verrucomicrobia* was found in contrast to the FM group. In general, there were more pronounced differences in species composition or variability in the species turnover of the FM-Nisin and FM-Q7 groups than that of the FM group.

**Figure 5:**
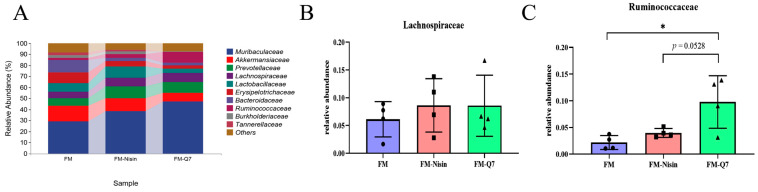
The relative abundance of different bacteria at the family level. (**A**) Microbial community bar plot by family; (**B**) *Lachnospiraceae*; (**C**) *Ruminococcaceae*. * *p* < 0.05.

Gut microbiome metabolites such as SCFAs modulated the percentage of beneficial and harmful bacteria in the gut microenvironment. It was reported that SCFAs were mainly derived from *Ruminococcaceae* and *Lachnospiraceae* [20,21]. After analyzing the differences between the two main acid-producing bacteria at the family level, it was found that the total occurrence of *Lachnospiraceae* and *Ruminococcaceae* was increased after the addition of nisin and plantaricin Q7 to fermented milk. The *Ruminococcaceae* in the FM-Q7 group was significantly increased (Figure 5C, *p* < 0.05). These results suggested that FM-Nisin and FM-Q7 could cause changes in the species abundance of host gut microbiota.

**Figure 6:**
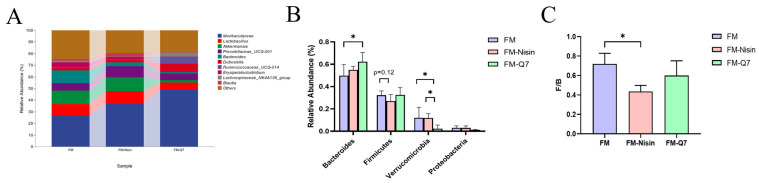
Different bacteria at the genus level. (**A**) Microbial community bar plot by genus. (**B**) The proportion of *Bacteroidetes*, *Firmicutes*, *Verrucomicrobia,* and *Proteobacteria*. (**C**) The ratio of *Firmicutes/Bacteroidetes*. * *p* < 0.05.

The analysis of genus-level species revealed *Bacteroides* occurrence was increased in the FM-Q7 group compared to the FM group. The abundance of *Firmicutes* in the FM-Nisin group and the abundance of *Verrucomicrobia* in the FM-Q7 group were remarkably less than that in the other two groups (Figure 6). Compared to the FM group, the F/B value of the FM Nissin group significantly decreased by 39.52% while the F/B value of the FM-Q7 group decreased by 16.59% (Figure 6C).

**Figure 7:**
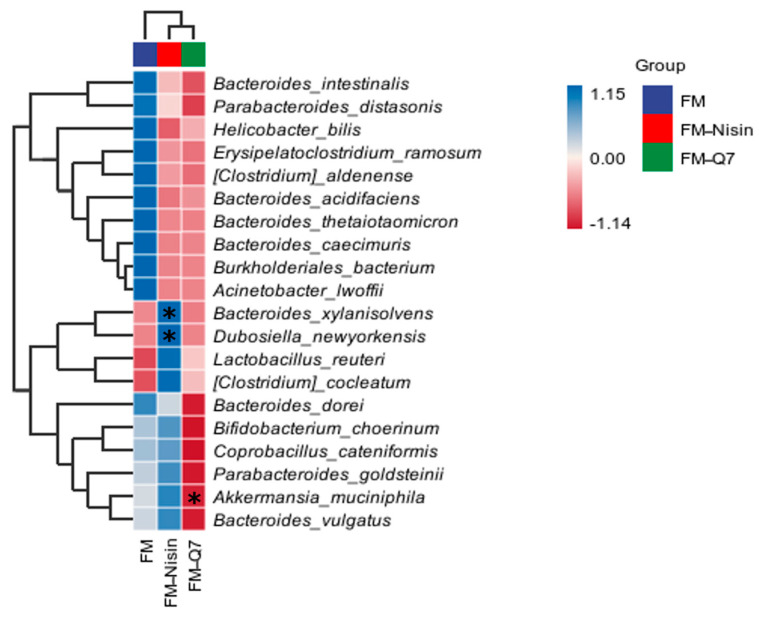
The abundance of different bacteria at the species level. * *p* < 0.05.

The effects of FM-Nisin and FM-Q7 on the richness of the host gut microbiota were investigated at the species level. In the FM-Q7 group, there was a significant decrease in *Akkermansia_muciniphila* occurrence while a notable increase in *Bacteroides_xylanisolvens* and *Dubosiella_newyorkensis* occurrence was observed (Figure 7). *Akkermansia_muciniphila* was found to comprise normal bacteria in the human gut and was representative of *Verrucomicrobia* [22], and often negatively correlated with the host inflammation level. This was consistent with the reduction in *Verrucomicrobia* occurrence and the low inflammation in the mice. The alterations in gut microbiota may comprise a potential factor in the regulation of the inflammatory stress level in the body. Interestingly, *Bacteroides_xylanisolvens* has emerged in recent years as an amazing treatment for NAFLD [23], and it was remarkably increased in FM-Nisin. This phenomenon indicated that the intake of FM-Nisin might lead to a change in the balance of gut microbiota.

**Figure 8:**
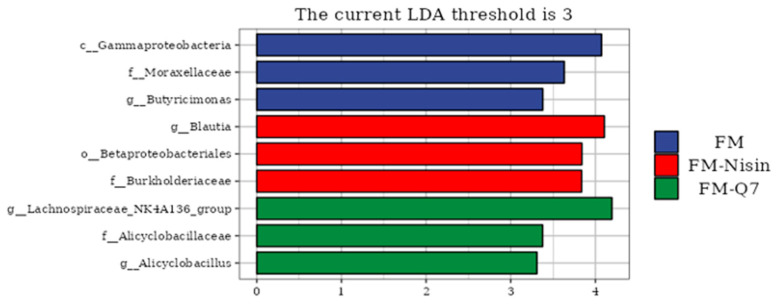
LEfSe analysis of bacterial communities at the genus level.

The LEfSe analysis results showed that compared with the group without FM, the marker species present in the host gut microbiota were significantly different after consuming FM-Nisin and FM-Q7. There was a minimal disparity in the enrichment of marker species between the FM-Nisin and FM-Q7 groups in comparison to the FM group (Figure 8). The *g_Lachnospiraceae_NK+4Al36_group* could improve the function of the intestinal barrier and produce butyrate, which was significantly negatively correlated with inflammation levels [24].

**Figure 9:**
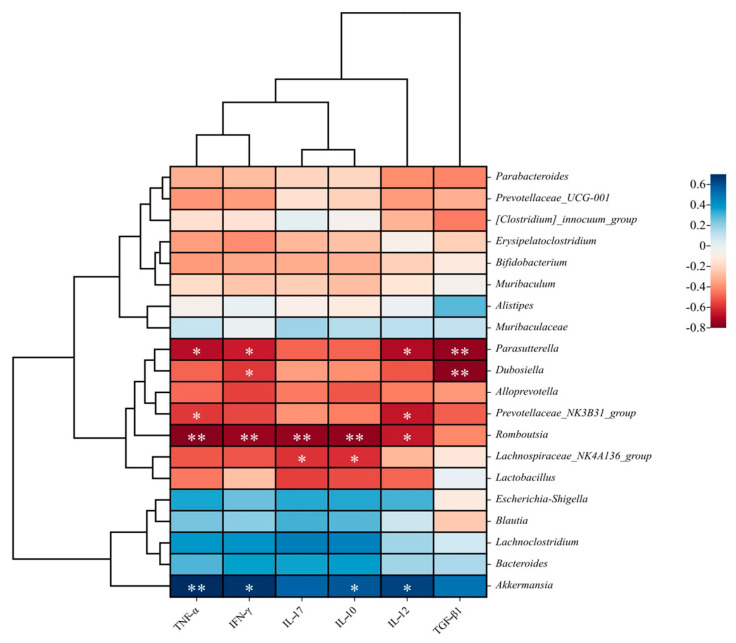
Correlation analysis between gut microbiota (genus level) and immuno-inflammatory factors. * *p* < 0.05, ** *p* < 0.01.

The effects of FM-Nisin and FM-Q7 on gut immunity and microbiota in mice were explored according to the report on Zhang et al.’s method [25]. Spearman correlation analysis was utilized to examine the correlation between gut microbiota and inflammatory factors. Gut microbiota play a vital role in intestinal inflammation. Different microbes participate in either pro-inflammatory or anti-inflammatory processes [26]. The consumption of fermented milk containing nisin and plantaricin Q7 was found to be positively linked to the presence of *Lachnospiraceae_NK4A136_group*, as evidenced by the low expression of IL-10 and IL-17 (*p* < 0.05). The butyric-acid-producing bacterium *Dubosiella* led to the down-regulation of IFN-γ and TGF-β1. There was a notable association between the expression of TNF-α and various bacterial genera including *Parasutterella*, *Prevotellaceae_NK3B31_group*, *Romboutsia*, and *Akkermansia*. The abundance of *Akkermansia* was significantly positively correlated with inflammatory factors TNF-α, IFN-γ, IL-12, and IL-10 after the consumption of fermented milk enriched with bacteriocins. The expression of the beneficial bacterium *Romboutsia* in mice was negatively correlated with serum inflammatory factors. In addition, correlation results with gut microbiome enrichment showed that the harmful bacterium *Parasutterella* was significantly negatively correlated with the expression of the anti-inflammatory factor TGF-β1 (Figure 9). Bacteriocins could subject a wide range of beneficial or harmful bacteria to the expression of inflammatory factors, and the presence of low-level inflammation in vivo might cause significant changes in the abundance and diversity of gut microbiota [13].

### 3.4. Effects of FM-Nisin and FM-Q7 on SCFAs in Mouse Colon Contents

**Figure 10:**
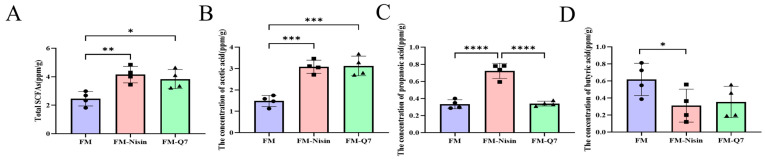
Effects of FM-Nisin and FM-Q7 on the content of SCFAs. Total short-chain fatty acids (**A**), acetic acid (**B**), propionic acid (**C**), and butyric acid (**D**). * *p* < 0.05, ** *p* < 0.01, *** *p* < 0.001, **** *p* < 0.0001.

SCFAs are considered beneficial gut microbial metabolites, and their levels can be quantified in mouse excrement [21]. The intake of FM-Nisin or FM-Q7 resulted in a significant increase in SCFAs content compared to the FM group (Figure 10A). Acetic acid and propionic acid content increased significantly after nisin intake (Figure 10B,C), and the butyric acid content decreased significantly (Figure 10D). The intake of plantaricin Q7 resulted in a significant increase in acetic acid content (Figure 10B) and a significantly lower propionic acid content than that in the Nisin group (Figure 10C), along with a decrease in butyric acid content (Figure 10D).

## 4. Discussion

Food preservatives are used widely to control microbial contamination in the food industry. Previously, food preservatives were believed to be biologically inert substances in the food system except for their antibacterial activity. Although, as per FDA classification, they are generally regarded as safe, food preservatives such as bacteriocins have been found to have a significant impact on gut microbiota and phenotypes. Consumers always ingest preservatives with food. In this study, we prepared three fermented milk samples including FM, FM-Nisin, and FM-Q7. Plantaricin Q7 is produced by *Lactiplantibacillus plantarum* Q7 and has more than 95% structural similarity to pediocin [16]. Umu et al. reported that class-II bacteriocins could regulate gut microbiota [11]. In our study, it was found that the appearance and body weight of the mice fed with FM-Nisin and FM-Q7 were normal, which was consistent with the generally recognized safe and non-toxic nature of biopreservatives. It was suggested that there were no obvious side effects of FM-Nisin and FM-Q7 on mice phenotypes. The expression levels of immune factors in the serum of mice were examined, and it was found that the expression of IL-17, IL-12, TNF-α, and IL-10 was significantly reduced in the FM-Nisin group. In general, there is a balance between pro-inflammatory and anti-inflammatory factors in the host. In our study, the expression level changes in IL-17, IL-12, TNF-α, and IL-10 in the FM-Nisin group might signify an immune response to the exogenous FM-Nisin. The effect of FM-Nisin on the inflammatory balance needs further study. In this study, the metabolic and immune systems of mice responded to exogenous nisin and plantaricin Q7 and alleviated the body abnormalities caused by inflammatory reactions. The host expressed various immune factors to prevent adverse reactions. This result was consistent with the reports of Chassaing et al. [27]. It was similar to the systemic low-grade inflammation induced by glycerol monolaurate in mice [28]. Interestingly, the alteration of gut microbiota and increased levels of gut SCFAs may be associated with the weak inflammatory response induced by FM-Nisin and FM-Q7.

There is growing evidence that gut microbiome disruption is closely related to the development of metabolic syndrome [29]. We further analyzed the effects of FM-Nisin and FM-Q7 on the composition of the gut microbiome at different biological levels. *Muribaculaceae* are major producers of acetic and propionic acids, and increasing their abundance is one way to mitigate inflammatory responses [30]. We found that microbial structures at the phylum, family, genus, and species levels of the FM-Nisin and FM-Q7 groups were different from those of the FM group. This was inconsistent with the statement of Gebhart et al. [8]. They observed that a bacteriocin specifically inhibiting *Clostridium difficile* did not affect gut microbiota diversity, which indicated that the effect of bacteriocins on gut microbiota had bacteriocin specificity. *Firmicutes* and *Bacteroidetes* are two major phyla of the domain bacteria in human gut microbiota. The ratio of *Firmicutes* to *Bacteroidetes* (F/B) has been extensively determined for human and mouse gut microbiota. When the occurrence of *Firmicutes* decreases or the occurrence of *Bacteroides* relative to *Firmicutes* increases, the F/B value decreases, and the body is at risk of chronic inflammation and digestive system diseases such as Crohn’s disease and ulcerative colitis [31]. The concentration of SCFAs in the FM-Nisin and FM-Q7 groups was significantly higher than that in the FM group. Our results further suggested that FM-Nisin and FM-Q7 could increase the levels of SCFAs, thereby attenuating injury in C57BL/6J mice.

There is an inevitable trend for food biological preservatives to replace chemical preservatives. Bacteriocins produced by lactic acid bacteria have potential as biological preservatives in dairy products and other foods [32]. Dairy products are rich in nutrients and susceptible to microbial contamination during processing and storage. To prolong shelf life and nutritional quality, the use of biological preservatives is indispensable. Nisin is recognized as a natural preservative in the food industry. Presently, all preservatives are subjected to a general toxicity test to determine the safety; we think that it is necessary to bring this into gut microbiota as an index of the safety evaluation, which is agreeable with the previous reports [32,33]. More and more studies have reported that food preservatives, sweeteners, silica nanoparticles, and bacteriocin might cause structural changes in gut microbiota and be associated with some metabolic disorders [34,35,36]. It has been confirmed that the gut microbiome is significant in maintaining host health and its close association with various chronic diseases such as inflammatory bowel disease, obesity, diabetes, and cardiovascular diseases [29,31]; therefore, it is reasonable to consider the stability and functionality of gut microbiota as a key criterion to evaluate the safety of food additives such as preservatives. Furthermore, in light of the global challenge posed by the abuse of antibiotics, bacteriocins are regarded as the ideal substitute for antibiotics. In addition to considering resistance, gut microbiome friendliness is significant in the development and application of novel bacteriocins to replace antibiotics.

In our study, our results suggested that the continuous consumption of preservatives over a short period might cause low-level inflammatory responses in mice. The short-lived mild inflammatory response was regulated by the autoimmune system and gut microbiota. Our study only explored the effects of fermented milk supplemented with nisin or plantaricin Q7 on inflammation and gut microbiota in mice. The existing results showed that the short-term intake of nisin or plantaricin Q7 could regulate the inflammation level and gut microbiota. Follow-up experiments need to be conducted on the effect of nisin or plantaricin Q7 on humans under long-term exposure.

## 5. Conclusions

The effects of FM, FM-Nisin, and FM-Q7 on inflammatory factors and gut microbiota were investigated in vitro. FM-Nisin and FM-Q7 could cause fluctuations in inflammatory cytokine levels in mice. Meanwhile, the species abundance of mouse gut microbiota significantly changed at the phylum, genus, family, and species levels. The analysis of the correlation between gut microbiota and inflammatory factors indicated that fluctuations in inflammation levels might be associated with an imbalance in the gut microbiota. The effect of fermented milk supplemented with nisin or plantaricin Q7 on the host’s health is required for further exploration.

## Figures and Tables

**Table 1:** Effects of FM-Nisin and FM-Q7 on mouse organ coefficient.

Item	FM (%)	FM-Nisin (%)	FM-Q7 (%)
liver	3.95 ± 0.40 ^b^	4.54 ± 0.84 ^a^	4.05 ± 0.36 ^ab^
kidney	1.11 ± 0.14 ^a^	1.09 ± 0.08 ^a^	1.06 ± 0.06 ^a^
spleen	0.36 ± 0.07 ^b^	0.46 ± 0.15 ^a^	0.36 ± 0.10 ^b^

^a,b^ Different letters indicate significant differences between groups (*p* < 0.05).

## Data Availability

The data presented in this study are available from the corresponding author.
